# Non-Invasive Voice-Based Early Detection of Parkinson’s Disease via Spectral Feature Analysis and Machine Learning Techniques

**DOI:** 10.3390/bioengineering13091026

**Published:** 2026-09-03

**Authors:** Yojhansen Omar Varela-Arellano, Manuel A. Soto-Murillo, Vanessa Alcalá-Ramírez, Karen E. Villagrana-Bañuelos, L. Rafael Salas-Rodriguez, Alejandra Cepeda-Argüelles, Ricardo Villagrana-Bañuelos, Jorge I. Galván-Tejada, Jose G. Arceo-Olague, Carlos E. Galván-Tejada

**Affiliations:** Unidad Académica de Ingeniería Eléctrica, Universidad Autónoma de Zacatecas, Jardín Juarez 147, Centro, Zacatecas 98000, Mexico; yojhansen.varelaa@uaz.edu.mx (Y.O.V.-A.); vdrar.06@uaz.edu.mx (V.A.-R.); kvillagrana@uaz.edu.mx (K.E.V.-B.); l.salas@uaz.edu.mx (L.R.S.-R.); acepeda@uaz.edu.mx (A.C.-A.); r.villagrana@uaz.edu.mx (R.V.-B.); gatejo@uaz.edu.mx (J.I.G.-T.); arceojg@uaz.edu.mx (J.G.A.-O.); ericgalvan@uaz.edu.mx (C.E.G.-T.)

**Keywords:** Parkinson’s disease, cepstral coefficients, speech processing, machine learning, vocal features

## Abstract

Background: Parkinson’s disease (PD) is a chronic, slowly progressive, and irreversible neuropathological disorder characterized by the progressive degeneration of specific neurons responsible for producing neurotransmitters essential for motor control. Although PD primarily affects motor function, various non-motor symptoms commonly emerge during the prodromal phase. These include autonomic dysfunction, cognitive and neurobehavioral disorders, and sensory and sleep abnormalities. Notably, speech and voice alterations, particularly hypokinetic dysarthria, are frequent manifestations. This research presents a methodology to distinguish between individuals with PD and healthy controls using voice signals through speech recognition and machine learning (ML) techniques. A dataset comprising 81 voice samples (41 healthy controls and 40 PD patients) was utilized to extract two types of cepstral features: Mel-frequency cepstral coefficients (MFCCs) and subband-based cepstral coefficients (SBCs). These extracted features were used to train and evaluate three ML algorithms: Random Forest (RF), K-Nearest Neighbors (KNN), and Support Vector Machines (SVM). Results: Among the algorithms evaluated, the SVM-SBC model exhibited the highest performance, achieving an accuracy of 79%, a sensitivity of 75.5%, and an Area Under the ROC Curve (AUC-ROC) of 84%. Conclusions: This study highlights the potential of integrating cepstral features with machine learning algorithms to develop reliable, non-invasive tools for the early detection of PD.

## 1. Introduction

Parkinson’s disease (PD) is a chronic, slowly progressive, and irreversible neuropathological disorder affecting the central nervous system (CNS) [1]. It is characterized by the progressive degeneration of specific neurons located in the basal ganglia, specifically within the substantia nigra pars compacta (SNpc) of the midbrain [2]. These neurons are responsible for producing various neurotransmitters, including dopamine, serotonin, and acetylcholine [3], which play a fundamental role in motor control. Consequently, PD predominantly affects motor function [4].

In particular, PD symptoms include both motor and non-motor manifestations. The primary motor signs, also known as cardinal clinical manifestations, include asymmetric resting tremor, bradykinesia, muscular rigidity, and postural instability. Conversely, non-motor symptoms primarily encompass autonomic dysfunction, cognitive and neurobehavioral disorders, and sensory and sleep abnormalities [5,6]. Additionally, speech and voice alterations are common manifestations, with hypokinetic dysarthria being one of the most frequent. This condition is part of a broad set of vocal emission impairments, including hypophonia, monotonous and monopitch speech, and hypokinetic articulation [7].

In clinical settings, PD diagnosis focuses primarily on identifying the motor syndrome [8]. Alongside clinical evaluation, neurologists utilize neuroimaging techniques, as well as genetic and biochemical tests [9]. However, the diagnostic process can be prolonged, taking an average of up to three years [10]. This delay occurs because the pathophysiological changes triggering PD symptoms are often not evident until at least four years after disease onset [11]. Unfortunately, characteristic motor symptoms typically appear only after significant degeneration has occurred, affecting between 50% and 60% of dopaminergic neurons in the substantia nigra and 60% to 80% of their striatal terminals [12]. This traditional diagnostic approach presents several limitations as it relies heavily on human intervention, which often leads to subjectivity issues. The skill and experience of the diagnosing physician directly influence the results, making these methods prone to error. Furthermore, the heterogeneity and complexity of PD characteristics hinder assessment. Symptoms vary between patients and do not always manifest in the same manner, order, or combination. The absence of overt motor or non-motor symptoms further increases diagnostic complexity, resulting in high inaccuracy rates [13]. Clinical diagnostic accuracy is estimated at approximately 80.6% (95% CI: 75.2–85.3%) [10]. The only conclusive diagnosis is histopathological analysis, which can only be performed post-mortem via autopsy [14]. These data suggest that a significant proportion of individuals with Parkinson’s may be undiagnosed or misdiagnosed [15].

To address these diagnostic limitations, recent advances in technology and computational capabilities have positioned artificial intelligence (AI) as a promising tool for supporting early diagnosis and disease prediction in healthcare [16]. In this context, the development of machine learning techniques, together with advances in speech processing, has enabled the creation of computational models for Parkinson’s disease detection based on acoustic voice features. Acoustic analysis of voice and speech can identify subtle patterns that are imperceptible during routine clinical assessment and detect changes in vocal parameters that may be used to predict PD progression and support clinical decision-making. Consequently, several studies have demonstrated that acoustic voice analysis holds considerable promise as an objective and non-invasive biomarker for Parkinson’s disease, facilitating earlier diagnosis, improving diagnostic reliability, and supporting continuous disease monitoring.

Although considerable progress has been achieved in speech-based Parkinson’s disease detection, most existing studies rely on conventional acoustic representations, particularly Mel-Frequency Cepstral Coefficients, owing to their proven effectiveness in speech processing applications. However, the exploration of alternative spectral parameterization techniques remains limited. In particular, Subband-Based Cepstral Coefficients, originally developed for automatic speech recognition, have received little attention in PD detection despite their ability to capture localized spectral characteristics that may better represent the subtle phonatory alterations associated with Parkinsonian speech. Consequently, it remains unclear whether SBCs can provide complementary or even superior discriminative information compared with conventional MFCC-based representations.

The present study proposes a methodology for the early and non-invasive diagnosis of Parkinson’s disease through acoustic voice analysis, specifically investigating the effectiveness of Subband-Based Cepstral Coefficients as acoustic descriptors for PD detection and systematically comparing their performance with conventional MFCCs, both individually and in combination. Additionally, Boruta-based feature selection (FS) and Bayesian hyperparameter optimization are incorporated to identify the most informative features and ensure a fair evaluation of classical machine learning classifiers.

The main contributions of this work are delineated as follows:

- Evaluation of SBCs as acoustic descriptors for PD detection: The application of subband-based cepstral coefficients (SBCs), originally developed for speech recognition tasks, is proposed as an alternative representation to MFCCs for PD detection using the sustained vowel /a/ phonation.
- Systematic comparison of spectral parameterization schemes: A comparative evaluation of two parameterization techniques is presented—MFCCs and SBCs individually, as well as the MFCC + SBC combination—under equivalent experimental conditions to determine the discriminative power of each representation.
- Feature selection using the Boruta algorithm: The Boruta algorithm is employed as a feature selection strategy to identify the most relevant acoustic descriptors within each parameterization scheme, reducing feature space dimensionality and improving model generalization.
- Bayesian hyperparameter optimization: Bayesian optimization is applied for the optimal configuration of KNN, SVM, and RF hyperparameters, ensuring a fair comparison and maximizing performance under each evaluated scheme.
- Comparative evaluation of classical ML classifiers: A systematic performance comparison of KNN, SVM, and RF is provided, contributing empirical evidence on which combination of acoustic descriptor and classifier offers the best balance between diagnostic accuracy, interpretability, and computational efficiency for early PD detection.

The subsequent sections of this paper are organized as follows: Section 2 provides a comprehensive overview of the current literature and related work; Section 3 describes the proposed methodology, including the feature extraction scheme and the classifiers employed; Section 4 specifies the experimental setup, implementation procedures, and reports the experimental results; Section 5 discusses the findings in the context of existing literature; and Section 6 highlights the main conclusions and suggests future research directions.

## 2. Related Work

Several studies have explored the implementation of machine learning models for PD detection through the analysis and processing of voice signals. Significant works addressing this topic are presented below.

Costantini et al. (2023) [17] investigated early PD detection by combining vocal feature analysis with machine learning and deep learning (DL) techniques. They constructed a custom dataset with high-fidelity recordings of vocal tasks obtained from 426 Italian subjects, of whom 160 were PD patients, distinguishing between early-stage patients without medication and mid-to-late-stage patients under L-Dopa treatment. Multiple ML pipelines with different feature selection and classification algorithms were compared, further exploring a custom CNN architecture. In the classification task of early PD versus healthy controls, SVM achieved the highest performdpimance among traditional classifiers, with an accuracy of 0.83, sensitivity of 0.83, specificity of 0.82, F1-score of 0.82, and AUC of 0.88, outperforming the CNN model, which obtained an accuracy of 0.70 and an AUC of 0.73. The results indicate that ML classifiers based on vocal features can match or even exceed DL models in early detection scenarios, highlighting feature selection via CFS as the most determinant factor in model performance.

Bukhari and Ogudo (2024) [18] proposed a PD detection system based on voice signals using an ensemble machine learning approach. They utilized the dataset [19] from the University of California, Irvine (UCI) repository, which incorporates vocal attributes such as time-frequency features, Mel-frequency cepstral coefficients, wavelet transform features, vocal fold features, and tremor waveform quality time. The model was built using the AdaBoost classifier, and its robustness was evaluated through cross-validation, demonstrating consistent performance across all iterations. The results reached an accuracy of 0.96, precision of 0.98, sensitivity of 0.93, F1-score of 0.95, and AUC of 0.99, demonstrating that the combination of spectral descriptors like MFCCs with wavelet transform features and tremor parameters in an ensemble classification scheme constitutes an effective strategy for early PD detection from voice signals.

More recently, in 2025, multiple research groups published relevant contributions that expanded the spectrum of methodological approaches in this field. Shen et al. (2025) [20] proposed a hybrid artificial intelligence model for early PD detection through voice analysis, combining convolutional neural networks, recurrent neural networks, multiple kernel learning, and multilayer perceptron. The study employed a dataset of 81 voice recordings [21], extracting acoustic features such as MFCCs, jitter, and shimmer for the classification of PD patients versus healthy controls. The hybrid model achieved an accuracy of 0.9111, sensitivity of 0.925, precision of 0.8984, F1-score of 0.9113, and AUC of 0.9125. For model interpretability, they incorporated SHAP values, which allowed for the identification of the most determinant vocal features in PD diagnosis. Additionally, they developed a probability-based scoring system aimed at monitoring disease progression.

Similarly, Naeem et al. (2025) [22] explored the potential of vocal biomarkers as prognostic indicators for early PD detection using ML techniques. The analyzed dataset [23] incorporated acoustic features such as fundamental frequency, jitter, shimmer, harmonics-to-noise ratio, and nonlinear dynamic complexity measures, obtained from 195 vocal recordings from 31 subjects. For preprocessing, they applied SMOTE to address class imbalance and Principal Component Analysis (PCA) as a feature selection strategy. Four classifiers were evaluated: SVM, Random Forest, Logistic Regression, and Decision Tree, using vocal measures to differentiate between PD patients and healthy individuals. Random Forest stood out as the most effective model, reaching an accuracy of 0.94 and a precision of 0.94, followed by SVM with an accuracy of 0.92 and a precision of 0.91. Upon incorporating PCA as a dimensionality reduction method, SVM obtained an accuracy of 0.89, while Random Forest and Decision Tree reached 0.87, respectively. The results underscore the relevance of vocal features combined with ML methods for reliable PD diagnosis, confirming that Random Forest and SVM are the highest-performing classifiers in voice-based binary classification tasks.

Xu et al. (2025) [24] developed an interpretable ML model for non-invasive PD detection from voice recordings. The study used the same dataset analyzed in [22] and incorporated SMOTE for handling class imbalance, splitting the dataset into 70% for training and 30% for testing. Multiple algorithms were evaluated, including KNN, SVM, Gradient Boosting, RF, and MLP. The Random Forest model achieved the best performance with an accuracy of 0.852, a sensitivity of 0.875 and an AUC of 0.936. For model interpretability, they employed SHAP values, which highlighted fundamental frequency variation and harmonics-to-noise ratio as the most relevant features for distinguishing PD patients from healthy individuals.

In the same vein, Rashidi et al. (2025) [25] developed a PD detection system based on a combination of long-term, short-term, and non-standard acoustic features, using the same dataset of 81 voice samples used in [20], composed of 41 healthy individuals and 40 PD patients. The extracted features included long-term descriptors such as jitter, shimmer, and cepstral peak prominence; short-term descriptors based on MFCCs; and non-standard measures such as pitch period entropy and recurrence period density entropy. Six ML algorithms were evaluated: RF, KNN, DT, Naïve Bayes, SVM, and LR, applying cross-validation to ensure the reliability of performance metrics. Random Forest was the highest-performing model, achieving an accuracy of 0.8272 and an AUC of 0.8965, followed by SVM with an accuracy of 0.7529 and an AUC of 0.8263, while KNN and Decision Tree obtained the lowest results among the evaluated classifiers. The authors highlighted that the combination of acoustic features of different temporal and spectral natures contributed to a more robust model for early PD detection compared to previous studies that used only a subset of descriptors.

Likewise, Quamar et al. (2025) [26] presented an automated system to distinguish between individuals with PD and healthy controls from voice signals, employing the same Figshare dataset [21] used in the present study. They extracted features such as MFCCs, spectrograms, Mel spectrograms, and waveform representations to capture characteristic vocal alterations of PD, including diminished vocal range, weak harmonics, elevated spectral entropy, and deteriorated formant structures. They evaluated traditional ML classifiers such as SVM, XGBoost, and LR, as well as deep learning architectures including DNN, CNN combined with LSTM, CNN with gated recurrent unit (GRU), and BiLSTM. Deep learning models outperformed the traditional ML classifiers, with BiLSTM being the highest performer with an accuracy of 0.97 and an AUC of 0.95, while the classic ML models achieved accuracies between 0.44 and 0.55.

Finally, Oumaima et al. (2026) [27] explored non-invasive PD detection through voice analysis and machine learning techniques. Their study employed the UCI dataset [23]. Eight ML algorithms were evaluated: XGBoost, RF, KNN, Gradient Boosting, AdaBoost, LR, SVM, and DT. As a preprocessing strategy, they applied SMOTE to address class imbalance followed by PCA for dimensionality reduction. XGBoost demonstrated the best performance with an accuracy of 0.9492, followed by Random Forest with 0.9322 and KNN with 0.8983, identifying Pitch Period Entropy, Recurrence Period Density Entropy, and spread1 as the most discriminative biomarkers.

A structured comparison of the reviewed studies is provided in Table 1, highlighting the employed datasets, acoustic features, classifiers, validation techniques, and reported results.

## 3. Materials and Methods

**Figure 1:**
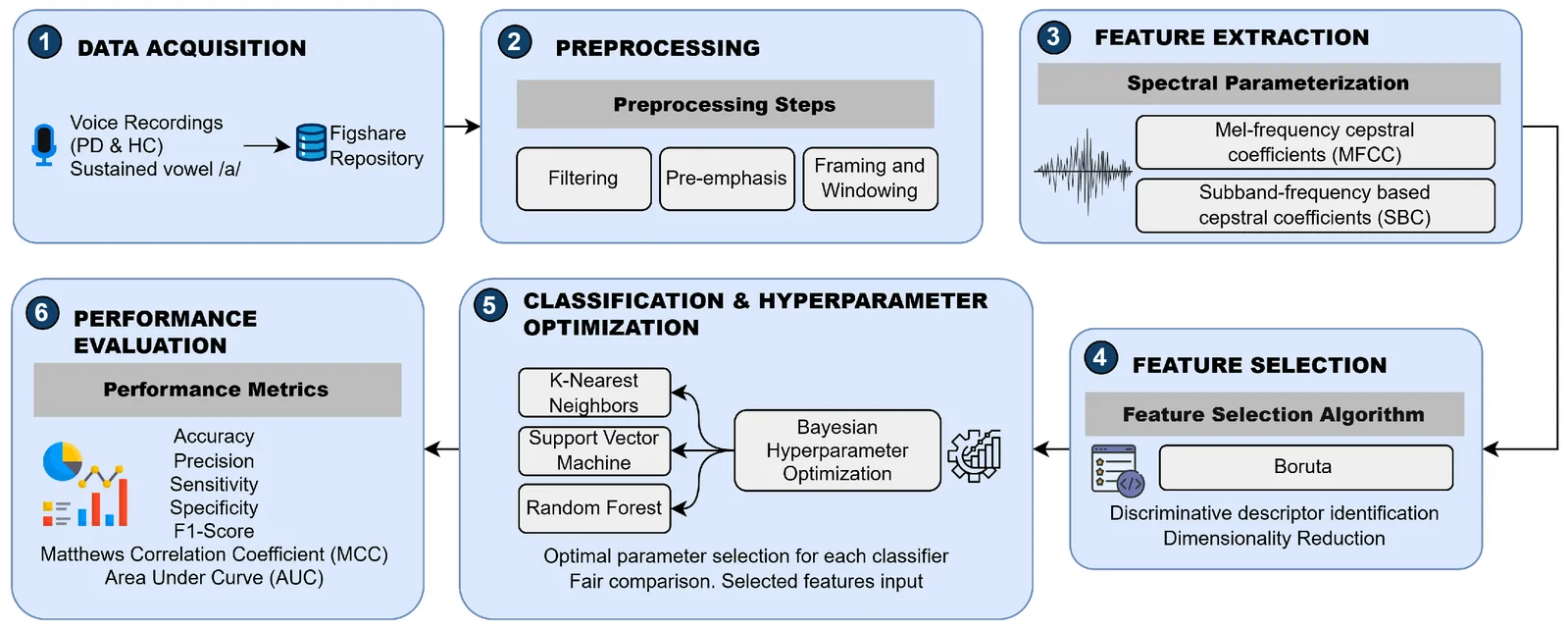
Flowchart of the proposed methodology.

Figure 1 provides a comprehensive end-to-end overview of the proposed classification framework. The workflow comprises six sequential stages that illustrate the complete methodological pipeline while highlighting the relationships between successive processing steps.

In the Data Acquisition stage (1), recordings were obtained from a publicly accessible dataset available in the Figshare repository, which contains voice records from PD patients and healthy individuals captured through sustained phonation of the vowel /a/. In Preprocessing (2), the recordings underwent filtering, pre-emphasis, framing, and windowing to ensure uniform input conditions for subsequent stages. During Feature Extraction (3), two spectral parameterization schemes were applied: Mel-frequency cepstral coefficients (MFCCs) and subband-based cepstral coefficients (SBCs). These were evaluated both individually and in combination to capture the spectral distortions associated with the hypokinetic dysarthria characteristic of PD. The Feature Selection stage (4) utilized the Boruta algorithm to identify the most discriminative descriptors within each parameterization scheme, reducing feature space dimensionality and improving model generalization. In the Classification stage (5), three supervised machine learning algorithms were implemented: K-Nearest Neighbors (KNN), Support Vector Machine (SVM), and Random Forest (RF), with hyperparameters optimized via Bayesian optimization. Finally, the Evaluation stage (6) assessed model performance using accuracy, precision, sensitivity, specificity, F1-score, Matthews correlation coefficient (MCC), and the area under the curve (AUC), selecting the configuration that achieved the highest global performance in binary classification between PD patients and healthy controls.

### 3.1. Data Acquisition

The dataset used in this study was obtained from a publicly accessible repository on Figshare [21]. It consists of voice samples from a sustained phonation task of the vowel /a/ lasting approximately three seconds. These samples were collected from 50 individuals with Parkinson’s disease (PwPD) and 50 healthy control (HC) subjects. The recordings were acquired using the participants’ own smartphones and subsequently sampled at 8 kHz. Following data cleaning, the final study population comprised 40 participants with PD and 41 healthy subjects. The demographic distribution of the participants is summarized in Table 2.

Based on this dataset, a preprocessing stage was conducted to prepare the voice signals for subsequent feature extraction.

### 3.2. Data Preprocessing

Voice signal preprocessing comprises several sub-stages, as outlined in the proposed methodology (Figure 1).

#### 3.2.1. Filtering

As previously mentioned, the dataset consists of recordings obtained via participants’ smartphones. Consequently, filtering is required to attenuate high-frequency components, such as interference or static, and to remove low-frequency noise, such as ambient hum or vibrations. A low-pass filter followed by a high-pass filter was applied to each voice signal.

The low-pass filter was implemented as a Butterworth IIR filter with a cutoff frequency of $F_{c}=3000\;\mathrm{Hz}$, while the high-pass filter was also designed as a Butterworth IIR filter with a cutoff frequency of $F_{c}=200\;\mathrm{Hz}$.

Applying low-pass and high-pass filters separately is preferred over using a single band-pass filter, as the latter may attenuate relevant components of the voice signal.

#### 3.2.2. Pre-Emphasis

In acoustic signal processing, pre-emphasis is deployed to balance the spectral energy distribution by compensating for the inherent high-frequency attenuation observed in human speech. This natural decay, commonly characterized as spectral tilt, primarily occurs due to the glottal source roll-off during phonation, combined with the physiological transfer characteristics of the vocal tract and radiation losses at the lips.

To mitigate this effect and flatten the speech spectrum prior to feature extraction, the signal is passed through a first-order high-pass Finite Impulse Response (FIR) digital filter. The operational behavior of this pre-emphasis stage is mathematically defined by the following *z*-domain transfer function [28]:

(1) $$H\left(z\right)=1-\alpha z^{-1}$$

where α represents the pre-emphasis coefficient that controls the magnitude of the high-frequency amplification, typically constrained within the interval 0.9≤α<1 [28].

Equivalently, the filter’s difference equation can be expressed as:

(2) $$S_{pp}\left[n\right]=S_{bp}\left[n\right]-\alpha \;S_{bp}\left[n-1\right]$$

where $S_{pp}\left[n\right]$ denotes the filter output signal, $S_{bp}\left[n\right]$ is the current input signal, and $S_{bp}\left[n-1\right]$ represents the previous sample. This filter attenuates low-frequency components and emphasizes rapid signal variations, increasing the contrast between adjacent samples [29]. In practice, particularly in cepstral coefficient extraction, a value of α=0.97 is commonly used and was adopted in this study.

#### 3.2.3. Framing and Windowing

The voice signal is a non-stationary random process, which poses challenges for analysis. However, this issue can be addressed by assuming that the signal is quasi-stationary over short time intervals. In framing, the signal is divided into short-duration segments (on the order of milliseconds), known as frames. Due to their brief duration (typically 10–30 ms) and low variability, the signal can be considered quasi-stationary within each frame. Adjacent frames typically overlap by one-third to one-half of the frame length to ensure smooth transitions.

Once the signal is segmented and the frames overlap, a windowing process is applied to reduce artifacts caused by abrupt changes at the frame boundaries. Common window types include rectangular, Hamming, and Hanning. Among these, the Hamming window is the most widely used in speech recognition, as its amplitude tapers gradually to a minimum value at the frame edges [30,31].

Mathematically, the Hamming window is defined by Equation (3):

(3) $$W_{hmm}\left(n\right)=0.54-0.46\cos\left(\frac{2\pi n}{N-1}\right),\;0\leq n\leq N,$$

where *N* denotes the number of samples in each frame. This window is applied to each overlapping frame of the voice signal.

In this stage, the previously filtered voice signals were segmented into 30 ms frames, a typical time interval over which the signal can be considered quasi-stationary. Each frame was overlapped by 50% and subsequently windowed using a Hamming window. Once processed, the windowed frames were stored in a matrix of dimension N×M, where *N* is the total number of frames and *M* is the number of samples.

### 3.3. Feature Extraction

This section introduces the theoretical background of Mel-frequency cepstral coefficients and subband-based cepstral coefficients, which constitute the basis of the proposed feature extraction strategy. It then describes the implementation adopted in this study, including the parameter settings and procedures used to generate each feature representation.

#### 3.3.1. Mel-Frequency Cepstral Coefficients

**Figure 2:**
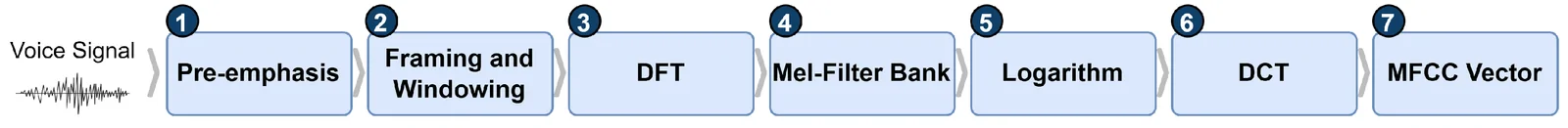
Flowchart of the MFCC feature extraction process.

MFCCs were introduced in 1980 by Davis and Mermelstein and are among the most widely used techniques in speech recognition and processing. The main idea of MFCCs is to model the human auditory system using a set of perceptual filters with center frequencies distributed non-linearly on an approximately logarithmic scale, resulting in a compact and robust representation for effective characterization [29,32,33]. Figure 2 illustrates the process of constructing an MFCC-based feature vector.

As shown in Figure 2, MFCC computation involves signal pre-emphasis, followed by framing and windowing. These stages are performed as part of the preprocessing.

Following this, each frame of *N* samples is transformed into the frequency domain using the Fast Fourier Transform (FFT), an efficient implementation of the DFT [31,32].

(4) $$X\left[k\right]=\sum_{n=0}^{N-1}x\left[n\right]\;e^{-j2\pi nk/N},\;0\leq k\leq N$$

Since X[k] contains both magnitude and phase information, the phase information is discarded, and only the spectral magnitude |X[k]| is retained [30].

Subsequently, the spectral magnitude is multiplied by a bank of *M* triangular filters spaced according to the Mel scale. This scale emulates the non-linear response of the human auditory system. The Mel scale relates the frequency *f* in Hz to the perceptual frequency through Equation (5) [34].

(5) $$\mathrm{mel}\left(f\right)=2595\cdot \log_{10}\left(1+\frac{f}{700}\right)=1127\cdot ln\left(1+\frac{f}{700}\right)$$

The Mel filter bank is defined by the following equations [32]:

(6) $$H_{m}\left[k\right]=\left\{\begin{array}{cc} 0, & k<f[m-1]\\ \frac{k-f[m-1]}{f[m]-f[m-1]}, & f[m-1]\leq k\leq f[m]\\ \frac{f[m+1]-k}{f[m+1]-f[m]}, & f[m]\leq k\leq f[m+1]\\ 0, & k>f[m+1] \end{array}\right.$$

where $H_{m}\left[k\right]$ denotes the response of the *m*-th triangular filter at frequency bin *k*, *m* is the filter index, and *k* is the discrete frequency bin index.

The logarithmic energy of each Mel filter is obtained by applying the filter bank to the power spectrum of the signal, followed by a logarithmic transformation:

(7) $$S\left[m\right]=\ln\left[\sum_{k=0}^{N-1}{\left|X\left[k\right]\right|}^{2}H_{m}\left[k\right]\right],\;0\leq m<M$$

where ${\left|X\left[k\right]\right|}^{2}$ represents the power spectrum of the signal.

Since the logarithmic energies of adjacent bands exhibit high statistical correlation, the DCT is applied to decorrelate the coefficients and project them onto the cepstral domain, yielding the MFCCs:

(8) $$MFCC\left[n\right]=\sum_{m=0}^{M-1}S\left[m\right]\cos\left[\frac{\pi n}{M}\left(m+\frac{1}{2}\right)\right],\;0\leq n<M$$

As a higher number of coefficients reflects faster variations in estimated energies and thus provides less useful information for signal classification, it is common to use between nine and thirteen coefficients, while higher-order coefficients are discarded [32,33].

#### 3.3.2. Subband-Based Cepstral Coefficients

Subband-based cepstral coefficients (SBCs) are obtained by applying the DCT to the logarithmic energy of subbands. In this regard, they are similar to MFCCs; however, the method used to decompose the signal into subbands differs.

Subband analysis employs a biorthogonal wavelet transform to perform a hierarchical time–frequency decomposition of the signal. This approach is based on the continuous wavelet transform, defined as the inner product of the signal x(t) with a family of wavelet functions $\psi_{a,b}\left(t\right)$, which are scaled (by *a*) and translated (by *b*) versions of the prototype wavelet ψ(t) [35]:

(9) $$\psi_{a,b}\left(t\right)=\psi \left(\frac{t-b}{a}\right)$$

(10) $$W_{\psi }x\left(a,b\right)=\frac{1}{\sqrt{a}}\int_{-\infty }^{+\infty }x\left(t\right)\psi^{*}\left(\frac{t-b}{a}\right)dt$$

**Figure 3:**
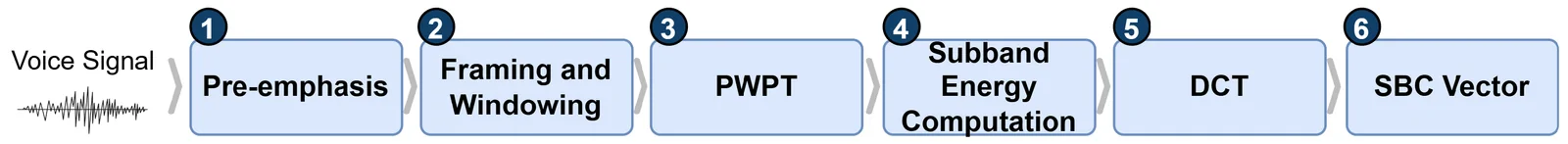
Flowchart of the SBC feature extraction process.

In its discrete-time implementation, the decomposition is performed through iterative two-channel filter banks, separating the signal into low-pass and high-pass components at each level, followed by a subsampling-by-two unit. Unlike the conventional wavelet transform, which iterates only on the low-pass branch, the wavelet packet filter bank tree can iterate on either branch at each level, generating a tree-like structure known as a wavelet packet filter bank tree [35]. Figure 3 shows the process of constructing an SBC-based feature vector.

The Perceptual Wavelet Packet Transform (PWPT) emphasizes low and medium frequencies by assigning a greater number of subbands to those regions. This decomposition approximately preserves a logarithmic distribution of subbands across the frequency spectrum [36].

Once decomposition is complete, the energy in each subband is calculated and scaled according to a determined number of coefficients for each band:

(11) $$E_{i}=\frac{\sum_{m\in i}{\left[\left(W_{\Psi }x\right)\left(i\right),m\right]}^{2}}{N_{i}}$$

where $W_{\Psi }x$ is the perceptual wavelet packet transform of the signal *x*, *i* is the frequency subband index, and $N_{i}$ is the number of coefficients in subband *i*.

Applying the DCT to the subband energies yields the SBCs. This is mathematically defined by Equation (12):

(12) $$SBC\left(j\right)=\sum_{i=1}^{M}\log E_{i}\cos\left[\frac{\pi j}{M}\left(i-\frac{1}{2}\right)\right]$$

for j=1,…,J, where *J* denotes the number of SBC coefficients and *M* the total number of frequency bands [37].

For both MFCCs and SBCs, 12 coefficients were computed. Six statistical descriptors were estimated for each coefficient: mean, standard deviation, skewness, kurtosis, minimum value, and maximum value. In addition, first- and second-order temporal derivatives (delta and delta-delta) were included to capture the temporal dynamics of the signal. These dynamic coefficients were computed using the following expression [38]:

(13) $$d_{t}=\frac{\sum_{n=1}^{N}n\left(c_{t+n}-c_{t-n}\right)}{2\sum_{n=1}^{N}n^{2}}$$

where $d_{t}$ denotes the delta coefficient at time *t*, estimated over a window of *N* adjacent frames.

### 3.4. Feature Selection

Feature selection (FS) is a fundamental process in machine learning that reduces the dimensionality of the feature space by eliminating redundant and irrelevant features, retaining only those most representative of the dataset.

In this study, feature selection was performed using the Boruta algorithm implemented via the BorutaPy package. This Random Forest-based wrapper method identifies relevant variables by comparing them with “shadow” attributes (randomized copies). The algorithm computes importance Z-scores and, using two-tailed hypothesis testing, retains only those features whose importance is significantly higher than the maximum Z-score of the shadow attributes (MZSA) [39]. This process is iteratively repeated until all variables are classified or a predefined iteration limit is reached.

The resulting subset of confirmed features was subsequently employed to train and evaluate the classification models.

### 3.5. Classification and Hyperparameter Optimization

Once the relevant feature subset was obtained using the Boruta algorithm, classification models were trained. Three classification algorithms were employed in this study, implemented in Python using the Scikit-learn library.

#### 3.5.1. Classification Models

- K-Nearest Neighbors (KNN): The KNN classifier is a non-parametric supervised learning algorithm that assigns a class label to an unknown sample based on the majority class among its *k* nearest neighbors in the feature space. Similarity between samples is measured using a distance function, with Euclidean distance being the most commonly employed. Its primary advantages are simplicity and interpretability; however, its performance is sensitive to the choice of *k* and the scale of input features, thus requiring proper data normalization prior to application [40].
- Support Vector Machine (SVM): SVM is a supervised classification algorithm that seeks the optimal separating hyperplane between classes by maximizing the margin between the support vectors of each class. For non-linearly separable problems, SVM employs kernel functions to project the data into a higher-dimensional space where linear separation is possible. Common kernels include linear, sigmoid, and radial basis function (RBF) [41].
- Random Forest (RF): RF is an ensemble learning method that constructs multiple decision trees trained on random subsets of data and features, combining their predictions through majority voting. Randomness is introduced via bootstrap sampling and random feature selection at each node, which reduces variance, decreases correlation among trees, and improves model generalization [42].

To optimize classifier performance, hyperparameter tuning was conducted to find the optimal configuration for each model. This procedure was performed using the BayesSearchCV function from the Scikit-Optimize library.

#### 3.5.2. Hyperparameter Optimization

Bayesian Optimization (BO) leverages prior information from previously evaluated parameter sets to determine the next set to assess. The method consists of two primary components: a probabilistic surrogate model that approximates the behavior of the objective function and the scalarization of this probabilistic model using an acquisition function to predict the next evaluation point. Over several iterations, BO builds a probabilistic model of the function being optimized, selecting the subsequent hyperparameter set based on the current best estimate of the function’s behavior, resulting in the optimal hyperparameter set for a machine learning model. By repeatedly updating this probabilistic model, BO systematically evaluates the hyperparameter space and converges toward the optimal set with a minimum number of function evaluations [43].

In contrast to the SVM and RF models, whose hyperparameters were optimized using Bayesian Optimization, the *k* hyperparameter of the KNN algorithm was tuned using Grid Search. The search spaces considered for each model are summarized in Table 3.

The search spaces were defined based on literature and practical considerations, providing a sufficiently wide range to explore optimal configurations without excessively increasing computational cost.

#### 3.5.3. Performance Evaluation Metrics

To obtain an unbiased estimate of the models’ generalization capabilities and strictly prevent information leakage, all model development procedures—including feature selection and hyperparameter optimization—were embedded entirely within a repeated nested cross-validation framework.

The outer loop consisted of a repeated stratified 5-fold cross-validation with three repetitions, used exclusively for model evaluation (approximately 80% of the samples for training and 20% for testing in each fold). Within each outer training partition, feature selection (Boruta), feature standardization (StandardScaler), and Bayesian hyperparameter optimization (BayesSearchCV) were performed independently. This rigorous separation ensured that no information from the test partitions influenced the model development process.

The performance of the resulting classifiers was assessed using several statistical metrics derived from the confusion matrix (Table 4), providing a comprehensive evaluation of the models’ classification capabilities:

The evaluation metrics considered in this study are summarized in Table 5. For each metric, the final performance is reported as the mean and standard deviation computed across all outer folds of the repeated nested cross-validation procedure.

In addition to the aforementioned metrics, the area under the ROC curve (AUC-ROC) was considered. The ROC (Receiver Operating Characteristic) curve is a graphical representation of the relationship between the true positive rate and the false positive rate across different decision thresholds. The AUC quantifies the area under this curve, providing a measure of the model’s ability to discriminate between classes, thereby evaluating model separability and the balance between sensitivity and specificity.

### 3.6. Implementation

The implementation, development, and coding of all stages of this research were performed using the Python programming language (v. 3.14.3). Experiments were conducted on a workstation equipped with an AMD Ryzen 7 processor (Advanced Micro Devices, Inc., Santa Clara, CA, USA), 32 GB of RAM, and the Windows 11 (64-bit) operating system.

## 4. Results

Following feature extraction, three acoustic feature datasets were generated: MFCC, SBC, and a hybrid representation (MFCC + SBC). The proposed classification framework was subsequently evaluated on these datasets using the repeated nested cross-validation strategy.

Within each outer training partition, Boruta feature selection and Bayesian hyperparameter optimization were performed independently to strictly prevent information leakage. Consequently, both the selected feature subsets and the optimized hyperparameters varied across the cross-validation folds, adapting dynamically to the specific training data of each iteration.

Therefore, rather than reporting a single feature subset or a unique hyperparameter configuration, the generalization capability of each classifier was assessed across all outer test folds. The final results are presented as the mean and standard deviation obtained from the complete repeated nested cross-validation procedure. To facilitate the analysis and interpretation of findings, the results obtained from each classifier were structured according to the characterization approach employed: MFCC, SBC, and the hybrid (MFCC + SBC) scheme.

### 4.1. MFCC-Based Approach Results

In this stage, model performance was evaluated using features extracted exclusively from MFCC. The detailed results are presented in Table 6.

**Figure 4:**
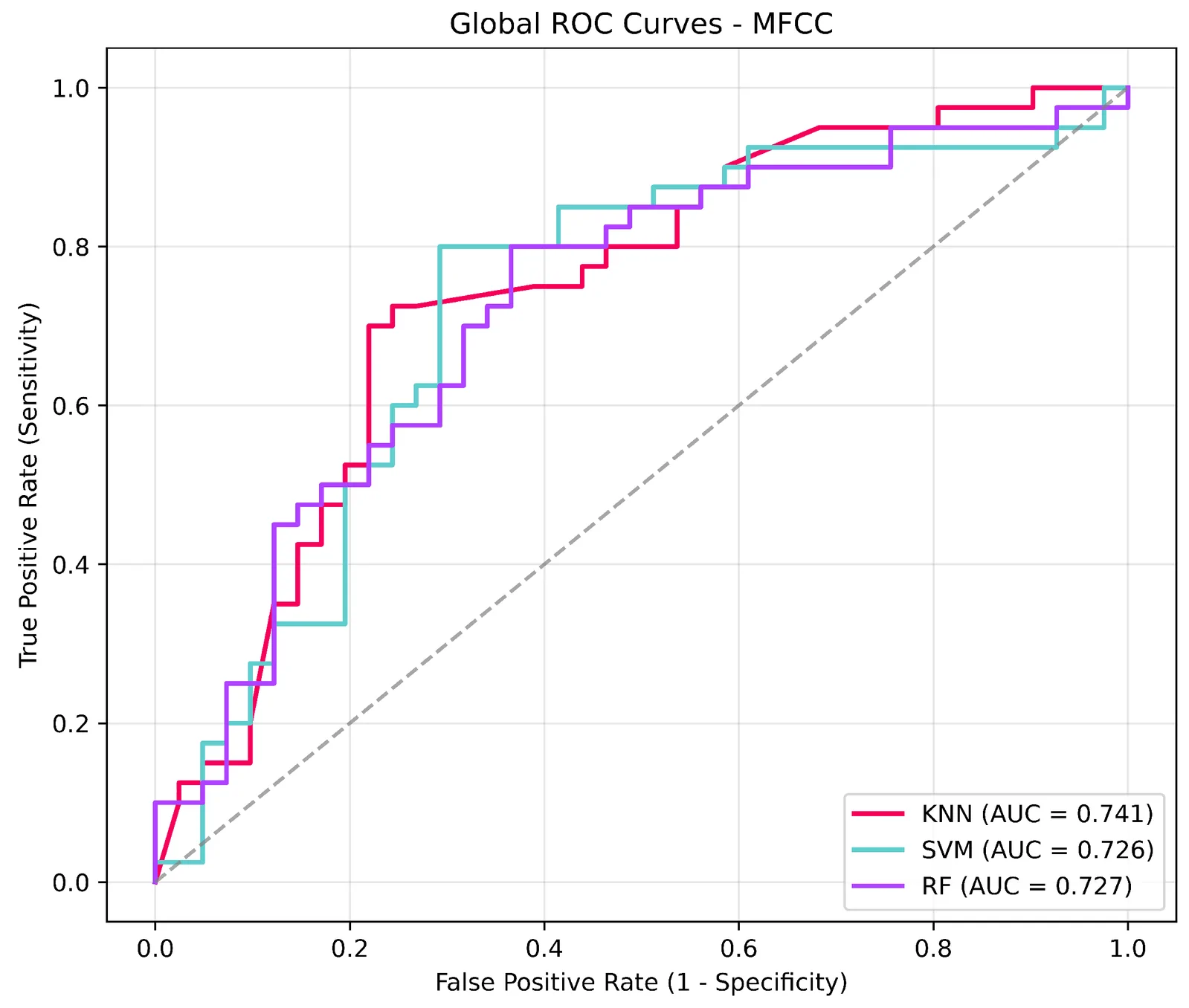
Receiver Operating Characteristic (ROC) curves for the classification models using the MFCC-based feature set. The area under the curve (AUC) represents the diagnostic capability of KNN, SVM, and RF classifiers. The dashed line represents the reference line corresponding to random classification performance.

According to the obtained results for the MFCC-based approach, the KNN model demonstrated the most robust overall performance, achieving the highest accuracy of 0.728±0.077 and an AUC of 0.741±0.078. In contrast, while SVM exhibited a peak specificity of 0.786±0.214, it suffered from a notably low sensitivity of 0.538±0.468 alongside substantial variance across folds, indicating instability in detecting positive cases. On the other hand, RF presented lower yet more stable and balanced predictive capabilities than SVM, which is evidenced by its remarkably low variance and an accuracy of 0.642±0.021. The models’ discriminative capability and the trade-off between true positive and false positive rates are illustrated by the ROC curves in Figure 4.

### 4.2. SBC-Based Approach Results

Subsequently, the subband-based representation was evaluated. As shown in Table 7, this approach yielded superior performance in PD detection compared to the MFCC framework across all classifiers.

**Figure 5:**
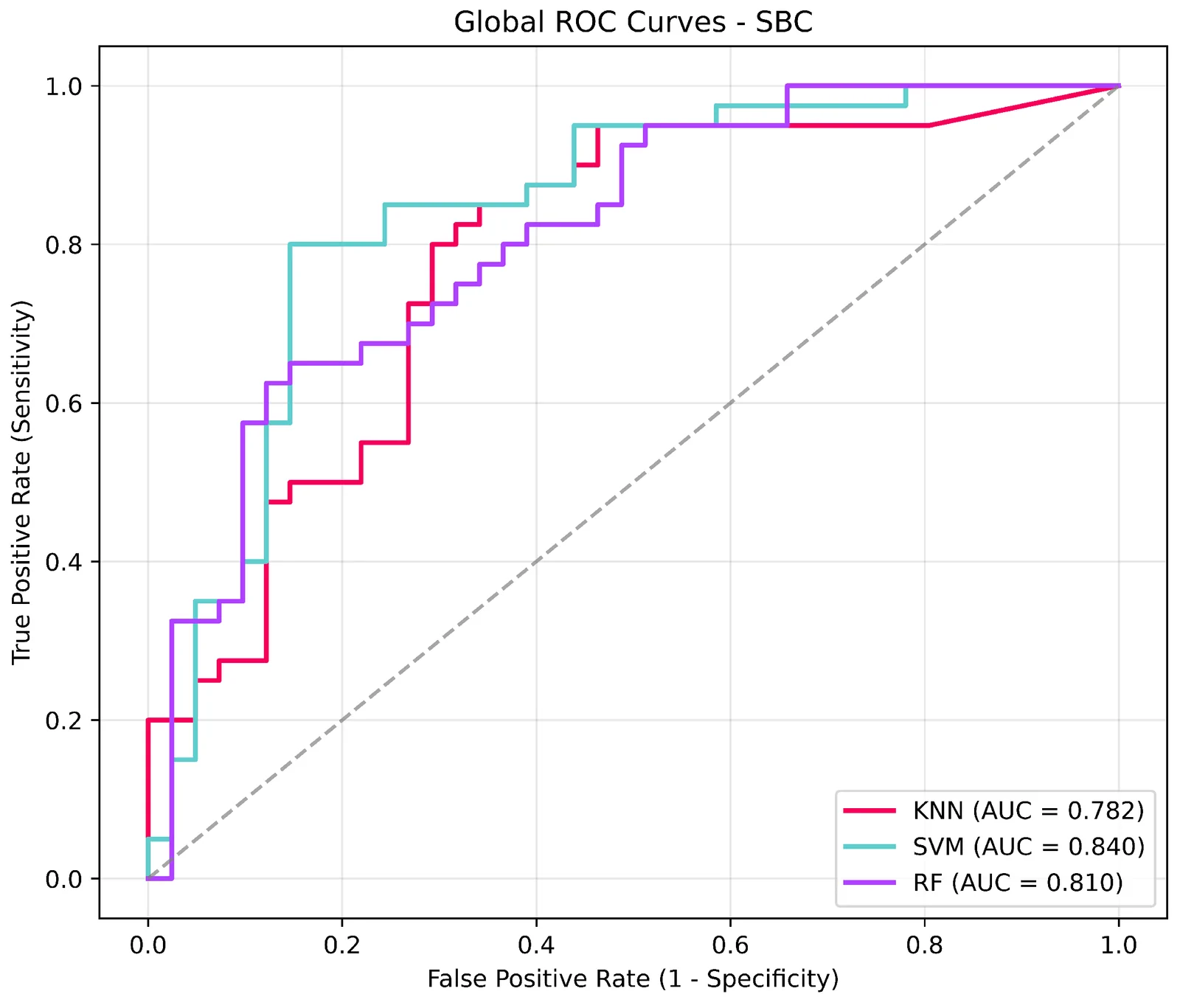
ROC curves for the classification models using the SBC-based feature set. The dashed line represents the reference line corresponding to random classification performance.

Through this approach, the SVM classifier stands out by achieving the highest accuracy of 0.790±0.093 and an MCC coefficient of 0.605±0.183, indicating a solid correlation between predictions and actual labels. This result is supported by the ROC curves in Figure 5, highlighting a peak AUC of 0.840±0.127 for the SVM model, which confirms its capability to distinguish between healthy subjects and PD patients within this feature representation.

### 4.3. Hybrid Approach Results (MFCC + SBC)

Finally, the integration of both feature extraction techniques was evaluated to assess whether a combined representation could improve system robustness. The resulting performance metrics for this hybrid (MFCC + SBC) approach are presented in Table 8.

**Figure 6:**
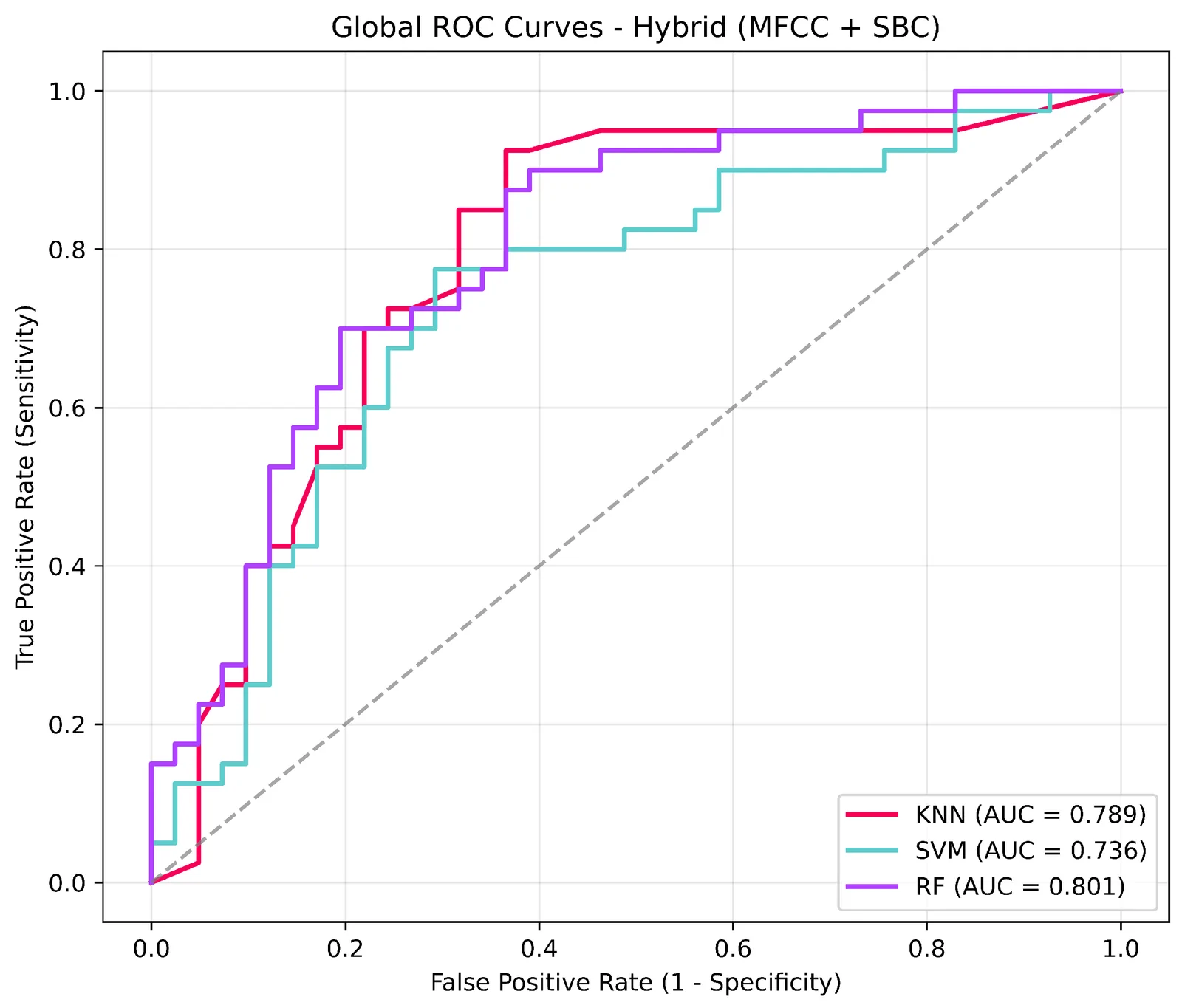
ROC curves for the hybrid approach. The dashed line represents the reference line corresponding to random classification performance.

Through this hybrid combination, the RF classifier stands out as the most balanced and robust model, achieving the highest accuracy of 0.753±0.057 and a remarkably high sensitivity of 0.875±0.042. While the integration of features did not strictly surpass the peak accuracy observed in the standalone SBC approach, it notably improved the sensitivity of the SVM model to 0.804±0.181 compared to its performance in previous representations. Furthermore, KNN maintained an excellent capability to identify healthy controls, yielding a peak specificity of 0.780±0.010 with exceptional stability across folds. This discriminative behavior and the overall trade-off between true positive and false positive rates are reflected in the ROC curves shown in Figure 6.

## 5. Discussion

The core objective of this study was to systematically evaluate whether localized multi-resolution spectral parameterization via Subband-Based Cepstral Coefficients could serve as a reliable, non-invasive biomarker for early Parkinson’s Disease detection using low-complexity machine learning classifiers. While our initial global optimization configuration yielded highly optimistic, near-perfect metric cross-sections (e.g., an initial standalone RF-SBC accuracy of 96% and an AUC of 0.98), subjecting the pipeline to a strict, leakage-free cross-validation infrastructure adjusted our top-performing model (SVM + SBC) to an honest, highly reproducible mean AUC of 0.840±0.127.

To formally validate the scientific positioning of this work, our unbiased results must be directly benchmarked against state-of-the-art studies that utilized the exact same Figshare open-access voice recording repository [21], as summarized in Table 9.

A critical analysis of contemporary literature reveals significant architectural vulnerabilities regarding data partitions and confounding variables. In the study conducted by Rashidi et al. [25], the authors integrated a massive combination of short-term (MFCC), long-term (jitter, shimmer), and non-standard nonlinear dynamics (PPE, RPDE) to achieve a Random Forest accuracy of 0.82 and an AUC of 0.89 using a standard 5-fold cross-validation. However, their feature selection and hyperparameter configurations were applied globally across the entire dataset. When global feature wrappers “see” the entire patient distribution prior to splitting, information regarding the validation partitions inherently leaks into the training domain. Our revised framework explicitly solves this flaw by embedding the Boruta wrapper uniquely inside the independent training loops of each fold, ensuring a true blind test. Similarly, Shen et al. [20] reported an accuracy of 0.9111 and an AUC of 0.9125 utilizing a heavy, highly complex hybrid deep learning infrastructure (CNN + RNN + MLP + MKL). While deep learning models natively isolate abstract representations, their validation relied on a single data split. On constrained cohorts (n=81), single data splits are highly unstable and prone to performance inflation. Furthermore, both Rashidi et al. [25] and Shen et al. [20] included demographic characteristics (such as age) directly into their classification features. Given that the mean age of the healthy control cohort is 47.73 years while the Parkinson’s cohort averages 67.08 years, models trained with age features inevitably learn to classify vocal senescence (aging) rather than pathological markers of Parkinsonian dysarthria. By completely purging age and sex from our feature matrices, our framework guarantees that our SVM-SBC mean AUC of 0.840 is genuinely disease-driven.

The most striking validation of our work emerges when contrasted with the deep learning framework published by Quamar et al. [26]. In their study, a Bidirectional Long Short-Term Memory (BiLSTM) network achieved an accuracy of 0.97 and an AUC of 0.95 using spectrogram representations. However, when Quamar et al. evaluated traditional, low-complexity machine learning algorithms under the same acoustic settings, their classical classifiers completely collapsed, yielding dismal accuracies bounded between 0.44 and 0.55.

This catastrophic drop highlights a profound limitation: standard acoustic parameterizations lack the descriptive power required by low-complexity classifiers when handling subtle voice pathologies. Our framework successfully breaks this limitation. By leveraging SBC descriptors via a Perceptual Wavelet Packet Transform, we achieved a stable classical SVM accuracy of 0.79 and an AUC of 0.84, outperforming Quamar’s classical baselines by up to 34% in accuracy. This proves that an engineered, neurophysiologically aligned feature extraction scheme can effectively compensate for the lower structural complexity of classical models. From a clinical and translational perspective, our framework presents a vital alternative for real-world deployment. While heavy deep learning architectures operate as uninterpretable "black boxes" that demand severe computational resources, a leakage-free SVM trained on targeted SBC features provides an energy-efficient, computationally light, and highly interpretable model that can be natively embedded into mobile health applications for remote, non-invasive telemonitoring and early clinical screening.

## 6. Conclusions

This research successfully demonstrates that voice alterations associated with Parkinson’s disease can be effectively modeled using classical machine learning architectures. This approach circumvents the need for computationally demanding deep learning models, provided that a highly localized spectral feature parameterization is utilized. By implementing a rigorous experimental pipeline completely free of data leakage and age-related confounding factors, this pilot study proves that subband-based cepstral coefficients offer a robust and statistically reliable diagnostic screening capability. The main scientific contribution of this work lies in demonstrating that precise, hand-crafted acoustic features aligned with the human auditory response—such as SBC—can significantly enhance the performance of classical classifiers. For instance, achieving a stable mean AUC of 0.840 with a SVM classifier elevates the predictive power well above the near-random baseline values ranging from 0.44 to 0.55 previously documented in the specialized literature [26].

Furthermore, since Parkinson’s disease is often diagnosed only after the onset of motor symptoms, leveraging non-motor manifestations like speech offers a promising, non-invasive approach for early detection. The experimental results indicate that developing computer-aided diagnostic systems from voice recordings is highly feasible and holds strong potential for integration into clinical settings and telemonitoring platforms. Importantly, these systems are not intended to replace the clinical judgment of medical specialists. Rather, they are designed to serve as objective, complementary tools that support medical decision-making, particularly during the early and prodromal phases where symptoms may be subtle and difficult to detect through conventional clinical evaluation.

Future research should focus on validating the proposed methodology across larger and more heterogeneous datasets. Acquiring a larger cohort would provide the sufficient statistical power required to perform formal pairwise statistical tests (such as the Wilcoxon signed-rank or permutation tests) across cross-validation folds, thereby further strengthening the comparative analysis of the classifiers. Furthermore, future work will aim to extend the acoustic analysis to a broader range of vocal tasks beyond sustained phonation. Additionally, incorporating SHAP (SHapley Additive exPlanations) values or permutation-based feature importance analysis will be explored to enhance model explainability, providing deeper clinical insights into the most discriminative vocal biomarkers.

## Figures and Tables

**Table 1:** Comprehensive overview of related works on voice and speech-based Parkinson’s disease detection.

Reference	Dataset	Acoustic Features	Model & Validation	Results
Costantini et al. (2023) [17].	Custom Italian dataset (426 subjects: 266 HC, 160 PD).	Jitter, shimmer, HNR/NHR, MFCC, vocal formants, DFA, RPDE, PPE, EMD-ER, GQ, GNE, and VFER.	ML: KNN, SVM, NB; DL: CNN. 10-fold CV.	SVM: Acc: 0.83, AUC: 0.88, Sen: 0.82, Spec: 0.82.
Bukhari & Ogudo (2024) [18].	Parkinson’s Disease Classification dataset (252 subjects: 64 HC, 188 PD).	TFF, MFCCs, Wavelet transform, VFF (jitter, shimmer, pitch), TWQT, and noise-related features.	ML: AdaBoost. Hold-out validation (80/20 train/test split).	AdaBoost: Acc: 0.96, AUC: 0.99, Sen: 0.93, Prec: 0.98.
Shen et al. (2025) [20].	Voice Samples for Patients with Parkinson’s Disease and Healthy Controls dataset (81 subjects: 41 HC, 40 PD).	MFCCs, jitter, shimmer, HNR, and pitch.	Hybrid Model: MLP + CNN + RNN + MKL. 5-fold CV.	Hybrid Model: Acc: 0.911, AUC: 0.912, Sen: 0.925, Prec: 0.898.
Naeem et al. (2025) [22].	Parkinson’s dataset (31 subjects: 8 HC, 23 PD).	Jitter, shimmer, pitch, RPDE, NHR, HNR, PPE, RPDE, D2 and DFA.	ML SVM, RF, LR, DT. Hold-out validation (80/20 train/test split).	RF (with SMOTE): Acc: 0.949, Sen: 1.00, Prec: 0.94.
Xu et al. (2025) [24].	Parkinson’s dataset	Same features used in [22].	ML: RF, LightGBM, SVM, LR, DT, NB, MLP, KNN, GB. Hold-out validation (70/30 train/test split).	RF: Acc: 0.852, AUC: 0.936, Sen: 0.875.
Rashidi et al. (2025) [25].	Voice Samples for Patients with Parkinson’s Disease and Healthy Controls dataset.	Long-term (jitter, shimmer, CPP), short-term (MFCC), and non-standard measures (PPE, RPDE).	ML: RF, KNN, DT, NB, SVM, LR. 5-fold CV.	RF: Acc: 0.827, AUC: 0.896, Sen: 0.750, Prec: 0.825.
Quamar et al. (2025) [26]	Voice Samples for Patients with Parkinson’s Disease and Healthy Controls dataset.	MFCCs, jitter, shimmer, HNR, RPDE, ZCR, CPP, RQA, GNE, pitch.	ML: SVM, XGBoost, LR; DL DNN, CNN + LSTM, CNN + GRU, BiLSTM. 10-fold CV.	BiLSTM: Acc: 0.97, AUC: 0.95, Sen: 0.98, Prec: 0.96.
Oumaima et al. (2026) [27].	Parkinson’s dataset.	Same features used in [22,24].	ML: XGBoost, RF, KNN, GB, AdaBoost, LR, SVM, DT.	XGBoost: Acc: 0.949.

**Table 2:** Demographics and clinical characteristics of the study population.

Label	No. of Participants	Mean Age	Std. Dev.	Age Range	Male	Female
HC	41	47.73	14.33	18–79	16	25
PwPD	40	67.08	9.01	43–85	21	19

**Table 3:** Hyperparameter search spaces for the classification models.

Models	Hyperparameter	Search Space
KNN	*k*	$\left\{3,5,\ldots ,\sqrt{n_{\mathrm{train}}}\right\}$ (Odd)
	*C*	$\left[10^{-3},100\right]$
SVM	γ	$\left[10^{-6},1.0\right]$
	Kernel	{rbf, sigmoid}
	n_estimators	[10,500]
	criterion	{gini, entropy}
	max_depth	[2,10]
RF	min_samples_split	[3,15]
	min_samples_leaf	[3,10]
	max_features	{sqrt, log2, None, 0.3, 0.5, 0.7}

**Table 4:** Confusion matrix.

	Predicted Positive	Predicted Negative
Actual Positive	TP	FN
Actual Negative	FP	TN

where TP (True Positives) represents correctly predicted positive instances; TN (True Negatives) corresponds to correctly predicted negative instances: FP (False Positives) denotes incorrectly predicted positive instances; and FN (False Negatives) indicates incorrectly predicted negative instances [44].

**Table 5:** Definitions and Mathematical Formulations of Performance Evaluation Metrics.

Metric	Description	Mathematical Definition
Accuracy	Measures the proportion of correct predictions relative to the total number of evaluated instances.	$\frac{TP+TN}{TP+TN+FP+FN}$
Precision	Measures the proportion of correctly predicted positive instances relative to the total number of predicted positive instances.	$\frac{TP}{TP+FP}$
Sensitivity	Quantifies the proportion of truly positive instances that are correctly identified as such.	$\frac{TP}{TP+FN}$
Specificity	Quantifies the proportion of truly negative instances that are correctly classified as such.	$\frac{TN}{TN+FP}$
F1 Score	Represents the harmonic mean of precision and sensitivity.	$\frac{2\cdot Precision\cdot Sensitivity}{Precision+Sensitivity}$
Matthews correlation coefficient	Provides a comprehensive measure of binary classification quality by considering all four values of the confusion matrix.	$\frac{TP\cdot TN-FP\cdot FN}{\sqrt{\left(TP+FP)(TP+FN)(TN+FP)(TN+FN\right)}}$

**Table 6:** Performance metrics of the classification models for the MFCC-based approach (mean ± standard deviation).

Metric	KNN	RF	SVM
AUC	0.741±0.078	0.727±0.030	0.726±0.008
Accuracy	0.728±0.077	0.642±0.021	0.654±0.167
F1-score	0.727±0.068	0.634±0.077	0.501±0.439
MCC	0.457±0.154	0.289±0.050	0.326±0.316
Precision	0.730±0.096	0.639±0.024	0.470±0.415
Sensitivity	0.725±0.040	0.648±0.164	0.538±0.468
Specificity	0.731±0.112	0.632±0.134	0.786±0.214

**Table 7:** Performance metrics of the classification models for the SBC-based approach (mean ± standard deviation).

Metric	KNN	RF	SVM
AUC	0.782±0.090	0.810±0.115	0.840±0.127
Accuracy	0.741±0.111	0.728±0.113	0.790±0.093
F1-score	0.764±0.100	0.754±0.090	0.779±0.103
MCC	0.498±0.241	0.474±0.223	0.605±0.183
Precision	0.692±0.070	0.701±0.126	0.835±0.150
Sensitivity	0.853±0.143	0.828±0.106	0.755±0.176
Specificity	0.632±0.088	0.632±0.196	0.830±0.179

**Table 8:** Performance metrics of the classification models for the hybrid (MFCC + SBC) (mean ± standard deviation).

Metric	KNN	RF	SVM
AUC	0.789±0.086	0.801±0.108	0.736±0.083
Accuracy	0.741±0.064	0.753±0.057	0.667±0.111
F1-score	0.726±0.075	0.780±0.033	0.704±0.091
MCC	0.485±0.120	0.525±0.106	0.373±0.236
Precision	0.755±0.024	0.706±0.069	0.641±0.112
Sensitivity	0.703±0.114	0.875±0.042	0.804±0.181
Specificity	0.780±0.010	0.632±0.134	0.535±0.223

**Table 9:** Comparative analysis with previous studies using the same Figshare dataset.

Study	Methods	Key Results
Rashidi et al. (2025) [25]	Long-term (jitter, shimmer, CPP), short-term features (MFCC), and non-standard measures (PPE, RPDE) using RF, KNN, DT, NB, SVM, and LR	RF: Acc (0.82), Sen (0.75), Prec (0.82), F1-Score (0.82), AUC (0.89)
Shen et al. (2025) [20]	MFCCs coefficients, jitter, and shimmer using a hybrid model combining CNN, RNN, MKL, and MLP	Hybrid deep learning model (MLP + CNN + RNN + MKL): Acc (0.91), Sen (0.92), Prec (0.89), F1-Score (0.91), AUC (0.91)
Quamar et al. (2025) [26]	MFCCs coefficients, Mel-spectrogram and standard spectrogram using ML models (SVM, XGBoost and LR) and Deep learning architectures (DNN, CNN + LSTM, CNN + GRU, BiLSTM )	BiLSTM: Acc (0.97), Sen (0.98), Prec (0.96), F1-Score (0.97), AUC (0.95)
Our research	MFCCs and SBC coefficients using KNN, SVM and RF	SVM + SBC: Acc (0.790±0.093), Sen (0.755±0.176), Prec (0.835±0.150), F1-Score (0.779±0.103), MCC (0.605±0.183), AUC (0.840±0.127)

## Data Availability

A publicly available dataset was analyzed in this study. The data can be found at: https://doi.org/10.6084/m9.figshare.23849127.v1 (accessed on 2 March 2026). The dataset is titled “Voice Samples for Patients with Parkinson’s Disease and Healthy Controls”.

## Abbreviations

The following abbreviations are used in this manuscript:

AI	Artificial Intelligence
AUC	Area Under the Curve
BO	Bayesian Optimization
DCT	Discrete Cosine Transform
DFT	Discrete Fourier Transform
DL	Deep Learning
FFT	Fast Fourier Transform
FS	Feature Selection
HC	Healthy Controls
KNN	k-Nearest Neighbors
MCC	Matthews Correlation Coefficient
MFCC	Mel-Frequency Cepstral Coefficients
ML	Machine Learning
PwPD	People with Parkinson’s Disease
PWPT	Perceptual Wavelet Packet Transform
RF	Random Forest
ROC	Receiver Operating Characteristic
SBC	Subband-Based Cepstral Coefficients
SVM	Support Vector Machine
